# A study of the efficacy of endoscopic cyclophotocoagulation for the treatment of refractory glaucomas

**DOI:** 10.4103/0301-4738.55062

**Published:** 2009

**Authors:** Seemant Raizada, Khalid Al Sabti

**Affiliations:** Al-Bahar Eye Center. Ibn Sina Hospital, Kuwait; 1 Faculty of Medicine. Kuwait University, Kuwait

Dear Editor

We read the article by Murthy *et al*.[1] with interest. We congratulate the authors for their study and agree with them regarding the utility of endoscopic cyclophotocoagulation (ECP) in refractory glaucoma. We have been using an endoscope for the past many years and have published reports for posterior[2] as well as anterior[3] endoscopic applications. We use microprobe E4 (manufactured by Endo-optiks Inc. Little Silver, NJ USA). We would like to suggest a few points to augment this fine study.

The authors present their data of 50 patients with refractory glaucoma, who underwent ECP by anterior or pars plana route. The aim of their study was “to evaluate the safety and efficacy of ECP in the management of refractory glaucomas”. The design of the study should have been more focused. ECP alone was done in only 12 (26.7%) cases. In all the remaining cases, ECP was done along with procedures like pars plana vitrectomy (PPV), silicone oil removal (SOR), endo-laser (EL), posterior chamber intraocular lens (PCIOL) implantation or anterior chamber intraocular lens (ACIOL) explantation. All these supplemental procedures can contribute to or show additive effect in decreasing intraocular pressure (IOP) or increasing best corrected visual acuity (BCVA). The authors should have judiciously chosen cases in which only ECP was done to comment specifically on the efficacy of ECP in refractory glaucoma.

In the study, all phakic eyes underwent anterior ECP. Even after inflating the ciliary sulcus, there is not much space between the iris and the anterior lens capsule. There is a high chance of inadvertent lens touch because we have to go near the ciliary processes. To preserve the intended laser energy setting, the optimal distance between the treated tissue and the endoscopic laser probe should be 2 mm.[4] Hence, the window of error is narrow. Moreover, presence of nuclear sclerosis or posterior subcapsular cataract limits the view through endoscope and increases the laser power needed for ciliary photocoagulation. We feel phakic eyes should undergo pars plana ECP, especially, with a curved endoscopic probe instead of straight probe to avoid posterior lens touch. Anterior approach ECP should be reserved for pseudophakic and aphakic cases.

Standard three-port vitrectomy is not needed in all cases of pars plana ECP. It is needed only if there is associated vitreous hemorrhage or retinal detachment which needs to be taken care of in the same sitting. In the majority of cases, two superior ports suffice. Irrigating light pipe can be used and only partial anterior vitrectomy is needed before inserting the endoscope inside the vitreous cavity. More sclerotomy openings and extra manipulations may cause more complications.

The authors conclude that “It (ECP) may offer better preservation of BCVA compared to the other trans-scleral methods of cyclodestruction”. This statement should be taken with a pinch of salt as in their study ECP was combined with phacoemulsification or PPV. The decrease in IOP or improvement in vision in their case series maybe also be due to supplementary procedures, hence ECP in this setting should not be compared to trans-scleral cyclodestruction.

## References

[1] Murthy GJ, Murthy PR, Murthy KR, Kulkarni VV, Murthy KR (2009). A study of the efficacy of endoscopic cyclophotocoagulation for the treatment of refractory glaucomas. Ind J Ophthalmol.

[2] Sabti KA, Raizada S, Kandari JA, Wani V, Gayed I, Kumar N (2008). Applications of endoscopy in vitreoretinal surgery. Retina.

[3] Al Sabti K, Raizada S, Al Abduljalil T (2009). Cataract Surgery Assisted by Anterior Endoscopy. Br J Ophthalmol.

[4] Yu JY, Kahook MY, Lathrop KL, Noecker RJ (2008). The effect of probe placement and type of viscoelastic material on endoscopic cyclophotocoagulation laser energy transmission. Ophthalmic Surg Lasers Imaging.

